# Improvement of performance of InAs quantum dot solar cell by inserting thin AlAs layers

**DOI:** 10.1186/1556-276X-6-83

**Published:** 2011-01-12

**Authors:** Dongzhi Hu, Claiborne CO McPheeters, Edward T Yu, Daniel M Schaadt

**Affiliations:** 1 Institut für Angewandte Physik/DFG-Center for Functional Nanostructures, Karlsruhe Institute of Technology (KIT), Karlsruhe, Germany; 2 Department of Electrical and Computer Engineering, Microelectronics Research Center, University of Texas at Austin, Austin, TX 78758, USA

## Abstract

A new measure to enhance the performance of InAs quantum dot solar cell is proposed and measured. One monolayer AlAs is deposited on top of InAs quantum dots (QDs) in multistack solar cells. The devices were fabricated by molecular beam epitaxy. *In situ *annealing was intended to tune the QD density. A set of four samples were compared: InAs QDs without *in situ *annealing with and without AlAs cap layer and InAs QDs *in situ *annealed with and without AlAs cap layer. Atomic force microscopy measurements show that when *in situ *annealing of QDs without AlAs capping layers is investigated, holes and dashes are present on the device surface, while capping with one monolayer AlAs improves the device surface. On unannealed samples, capping the QDs with one monolayer of AlAs improves the spectral response, the open-circuit voltage and the fill factor. On annealed samples, capping has little effect on the spectral response but reduces the short-circuit current, while increasing the open-circuit voltage, the fill factor and power conversion efficiency.

## Introduction

Group III-V compound semiconductor solar cells are the highest efficiency cells developed to date [1] due to the wide range of bandgaps that can be grown with high crystalline quality in this material system. Record efficiencies around 40% [2] are achieved in triple junction cells with an InGaP/InGaAs/Ge structure, in which lattice-matched InGaAs replaces a middle GaAs layer. Due to the three-dimensional confinement of quantum dots (QDs), their incorporation into the middle layer enhances the photo current of solar cells, which can be further improved by forward scattering techniques [3]. The electronic properties of QDs depend on their size, shape, and surrounding matrix [4] and can be tuned during molecular beam epitaxy (MBE) by growth rate, temperature, and *in situ *annealing procedures [5,6]. In particular, it is possible to tailor the absorption spectrum of the InAs QDs to the 1.0 to 1.2 eV range, which allows for enhanced absorption with respect to the solar spectrum. To tailor the absorption spectrum of the InAs QD, an *in situ *annealing procedure is often used. Annealing of InAs QDs at relatively low temperatures, i.e., lower than 470°C right after their deposition leads to classical Ostwald ripening. In this case, the dot density decreases with smaller dots disappearing while larger dots growing with annealing time. When the dot size becomes larger than a critical value, dislocations are formed, which is not preferred for solar cells. However, when annealing InAs QDs at relatively high temperatures, i.e., higher than 490°C, a combination of ripening and InAs decomposition occurs. The size and chemical composition of QDs can then be tuned without formation of defects [7]. For enhancement of absorption, vertical stacks of InAs QDs embedded in InGaAs/GaAs are preferred, where segregation of In has to be considered, especially with an *in situ *annealing procedure [8]. To suppress this In segregation, a thin AlAs capping layer can be introduced [9,10]. However, the effect of this thin AlAs layer on the device performance is thus unclear and needs to be studied.

In this study, we fabricated GaAs-based *p-i-n *junctions with a stack of 10 layers of InAs QDs as the intrinsic layer. The density of the InAs QDs in each layer was tuned by *in situ *annealing at high temperature. To suppress In segregation and thereby keeping the InAs QD composition approximately constant, one monolayer AlAs was deposited on top of the QD layer. The influence of capping the dots with a monolayer of AlAs on devices with and without *in situ *annealed QDs is investigated.

## Experimental

The solar cell structures are shown in Table 1. Before growth of the structures, epi-ready Si-doped GaAs (100) substrates were pre-degassed in a load lock chamber at 130°C for 1 h. Then the substrates were transferred into the growth chamber and heated up to 600°C under As_4 _ambient conditions to remove the oxide layer. *In situ *reflection high energy electron diffraction (RHEED) was used to observe the surface reconstructions. When a clear 2 × 4 reconstruction appeared, we decreased the temperature to 570°C to start growth of a 1 μm thick n-doped GaAs with a doping level of 7 × 10^18 ^cm^-3^. Then 10 stacks of 20 nm intrinsic GaAs, covered by InAs QDs capped with a 6.6 nm thin In_0.12_Ga_0.88_As layer, were deposited. For the InAs QDs layers, we deposited nominally 2.3 monolayers, which correspond to 0.69 nm thick. Since the InAs QDs are formed in the Stranski-Krastanov growth mode, this leads to a wetting layer of about 1.6 monolayer thickness and the QDs on top of it. For devices with *in situ *annealed QDs, the growth was interrupted right after deposition of each of the InAs QDs for 5 min. The temperature was kept at the growth temperature, thereby allowing for In surface diffusion and desorption, resulting in a combined rearrangement of the InAs QDs to a distribution with lower dot density and lower In content [7]. For devices with AlAs capping, one monolayer (0.283 nm) of AlAs was deposited on top of the QDs before covering them with the 6.6 nm thick In_0.12_Ga_0.88_As layer. At the end a 20 nm thick intrinsic GaAs layer followed by a 100 nm thick p-doped GaAs of 1 × 10^19^cm^-3 ^was deposited. Silicon and Beryllium were used as n- and p-type dopants. During growth, the equivalent beam pressure of As_4 _remained at 3.0 × 10^-6 ^Torr and equivalent beam pressure of Ga, In, and Al were 4.67 × 10^-7 ^Torr, 2.0 × 10^-8 ^Torr, and 1.2 × 10^-8 ^Torr, respectively. The temperature was calibrated by a pyrometer. The growth rates were calibrated by RHEED specular spot oscillations. In total, we fabricated four types of samples: sample A contains unannealed QDs without AlAs capping. Sample B contains unannealed QDs with AlAs capping. Samples C and D contain annealed QDs without and with AlAs capping, respectively. In order to distinguish the difference of these four types of samples, they are summarized in Table 2. The morphology of the device surface was measured by atomic force microscopy (AFM) in contact mode. Devices were illuminated with a Newport Oriel 96000 solar simulator operating at 160 W while device performance was measured with a Hewlett-Packard 4156A analyzer. More details on fabrication of contacts and photocurrent measurements are given elsewhere [11].

**Table 1:** Layer structures for samples without and with AlAs capping

Without AlAs capping		With AlAs capping	
100 nm p-GaAs		100 nm p-GaAs	

20 nm i-GaAs		20 nm i-GaAs	

6.6 nm i-InGaAs		6.6 nm i-InGaAs	
InAs QDs	10×	0.283 nm AlAs	10×
		InAs QDs	
20 nm i-GaAs		20 nm i-GaAs	

1 μm n-GaAs buffer		1 μm n-GaAs buffer	

n-GaAs substrate		n-GaAs substrate	

**Table 2:** Four types of samples

	Sample A	Sample B	Sample C	Sample D
With InAs QD layers	As grown	As grown	Annealed for 5 min	Annealed for 5 min
QDs are covered by 1 ML AlAs layers	No	Yes	No	Yes

## Results and discussion

**Figure 1:**
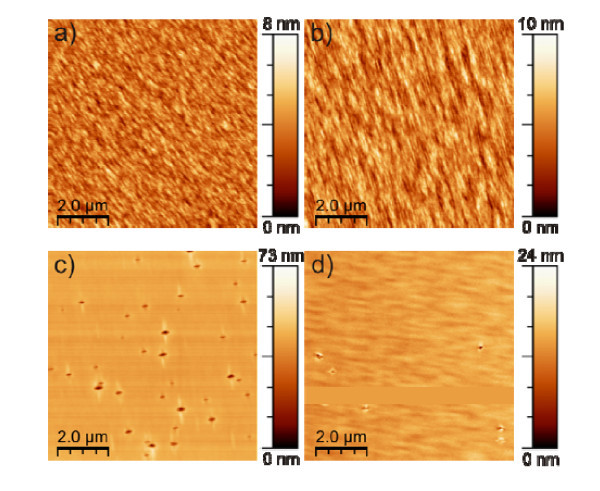
**AFM images of device surfaces**. **(a) **With as-grown InAs QDs and without AlAs layers (sample A), **(b) **with as-grown InAs QDs and AlAs layers (sample B), **(c) **with annealed InAs QDs and without AlAs layers (sample C), and **(d) **with annealed InAs QDs and AlAs layers (sample D).

Figure 1 shows AFM images of all four samples. The surface of samples A and B appears similar. For sample A, the RMS roughness is 1.12 nm over an area of 10 × 10 μm^2^. It is slightly better than the RMS roughness of sample B, which is 1.16 nm for an area of 10 × 10 μm^2^. From the AFM image of sample C, which contains annealed InAs QD layers, dashes and holes are clearly visible. The holes are anisotropic with the dashes running perpendicular to the elongated directions of the holes. However, AFM images of sample D, which consists annealed QDs covered by 1 ML AlAs, show that a much lower density of holes is present and the dashes are barely visible compared to sample C. The scale bars of AFM images of sample C and D indicate that the holes are much deeper on sample C than the ones on sample D, although the scales do not show the real values due to the limitation of the AFM tip. The appearance of holes and dashes on sample C can be explained as a result of decomposition of InAs, In segregation and In-Ga intermixing [7], as well as Ga(In) migration anisotropically along the [1-10] and [110] direction during the *in situ *annealing procedure[12]. These processes lead to a roughening of the surface which accumulates after 10 periods of annealed QDs. These effects are however suppressed or strongly reduced by inclusion of the AlAs capping layer, as seen in sample D. Especially the formation of holes could be explained as same as the formation of nanostructures by droplet epitaxy [13].

**Figure 2:**
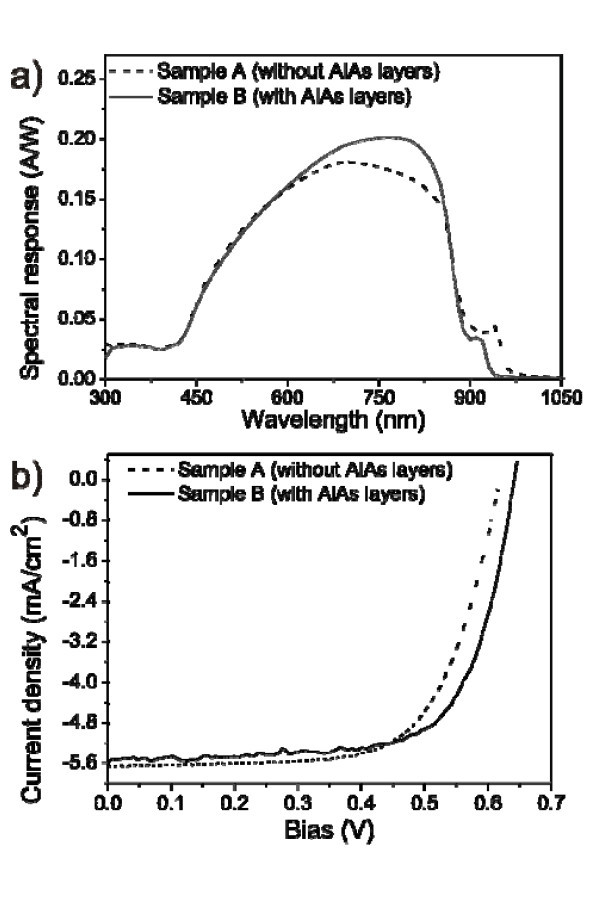
**Photocurrent spectra and current density-voltage (*J-V*) curves of devices with as-grown QDs with (sample A) and without AlAs capping layers (sample B)**. **(a) **Photo-current spectra and **(b) ***J-V *curves.

**Figure 3:**
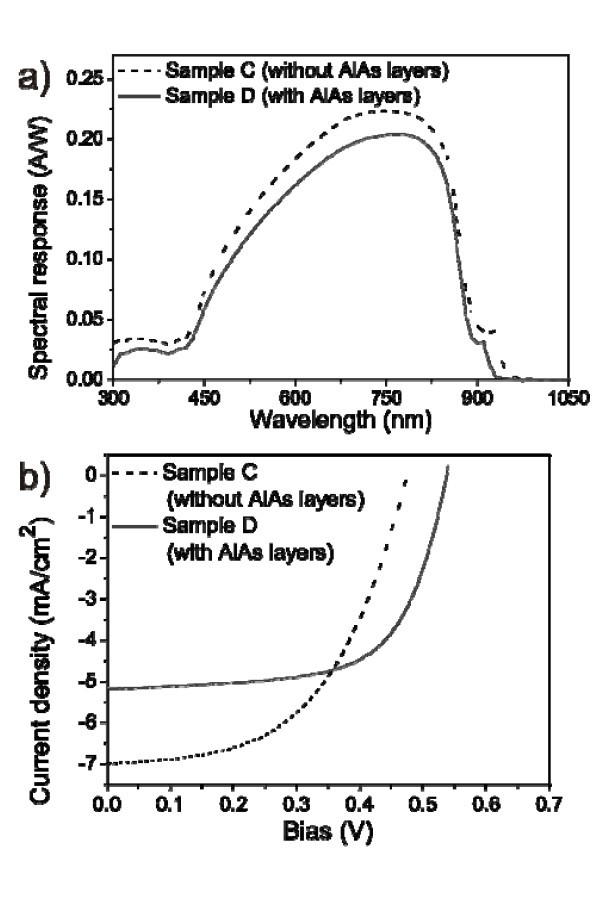
**Photocurrent spectra and current density-voltage (*J-V*) curves of the devices with annealed QDs with (sample C) and without AlAs capping layers (sample D)**. **(a) **Photo-current spectra and **(b) ***J-V *curves.

Figure 2a shows the room temperature photocurrent spectra of samples A and B. The response peak of QD layer from sample A is at longer wavelength comparing to the response peak of sample B. This is consistent with the studies of Suzuki et al. [14]. The intermixing of InAs with GaAs can be suppressed by inserting a thin AlAs layer between InAs and GaAs. Therefore, the response wavelength from QDs covered by AlAs blue-shifts compared to the response wavelength of the sample without AlAs layers. The same occurs for the devices with annealed QDs, i.e., for samples C and D, as shown in Figure 3a.

The curves of current density versus voltage (*J*-*V*) of samples A and B are shown in Figure 2b, while those for samples C and D are shown in Figure 3b. The fill factors, illumination short-circuit current densities (*J*_sc_), open-circuit voltages (*V*_oc_), and maximum power density (*V*_M_*J*_M_) are summarized in Table 3. The fill factor is calculated according to the equation:

$$\text{FF}=(V_{M}J_{M})/(V_{OC}J_{SC}).$$

**Table 3:** Summary of short-circuit current density, open-circuit voltage, fill factor, and maximum power density for samples A (as-grown QDs without AlAs layer), B (as-grown QDs with AlAs layer), C (annealed QDs without AlAs layer), and D (annealed QDs with AlAs layer)

	Sample A	Sample B	Sample C	Sample D
Short-circuit current density (mA/cm^2^)	-5.65	-5.56	-7.00	-5.26
Open-circuit voltage (V)	0.617	0.642	0.475	0.540
Fill factor	0.671	0.696	0.524	0.631
Maximum power density (*V*_M_*J*_M_)	2.340	2.484	1,743	1,791

*J*_sc _for all the samples are similar except for sample C. As seen from the AFM images in Figure 1, sample C has deep holes on the surface. Their effect on the device performance is thus far unclear and is currently under investigation. From Table 3, one can see that the open-circuit voltages of the samples (samples A and B) with as-grown InAs QDs have higher values. Also, for the samples with one monolayer AlAs layers (samples B and D), the open-circuit voltages are improved compared to samples without one monolayer AlAs capping layers (samples A and C). Looking at the fill factors and open-circuit voltages, one can conclude that both quantities are higher for samples with AlAs layers than those of samples without AlAs layers, no matter with as-grown QDs or with annealed QDs. The reason for improvement of open-circuit voltages and fill factors in samples with a thin AlAs layer is currently under investigation. Furthermore, by calculation of the power conversion efficiency (*η*) with the equation:

$$\eta =V_{M}J_{M}/P(P\;\text{is}\;\text{the}\;\text{illumination}\;\text{power}\;\text{density}),\;$$

we can conclude that with thin AlAs layers, the efficiency is enhanced by 6.15 and 2.75% for the devices with as-grown QDs and annealed QDs, respectively.

Comparing Figures 2 and 3, one can see that the solar response spectra of samples C and D with annealed QDs are blue-shifted compared to those for samples A and B. The photo currents and open-circuit voltages of these samples with annealed QDs decrease. The blue-shift of the solar response spectra is probably due to the change of InAs composition, which decreases during annealing [7]. The decrease of photo currents can be related to the lower QD density due to *in situ *annealing. A detailed explanation for this decrease in QD density during *in situ *annealing can be found in [7]. The holes and dashes are most likely the major reason for lower open-circuit voltage of the device with annealed QDs.

## Conclusions

GaAs-based *p-i-n *solar cells with a multistack of InAs QD embedded in InGaAs/GaAs matrix were fabricated by MBE. Post-growth annealing was used to tune the InAs QDs density. Annealing of InAs QDs introduces holes and dashes on the device surface and the solar response spectrum is blue-shifted, while the fill factor decreases. With insertion of one monolayer AlAs capping on top of the annealed InAs QDs for each layer in the stack, the dashes on the surface disappear and the density of holes decreases. The fill factors and power conversion efficiency and open-circuit voltages are improved, although the short-circuit current is reduced.

## Abbreviations

AFM: atomic force microscopy; MBE: molecular beam epitaxy; QDs: quantum dots; RHEED: reflection high energy electron diffraction.

## Competing interests

The authors declare that they have no competing interests.

## Authors' contributions

DZH carried out growth of the devices and COMcP carried out electronic characterization of the devices. ETY and DMS conceived of the study, and participated in its design and coordination. All authors read and approved the final manuscript.
